# Early CT Ameliorates the Diagnostic Dilemma of “Little Old Lady’s Hernia”: A Case Report

**DOI:** 10.31729/jnma.7499

**Published:** 2022-08-31

**Authors:** Subodh Ghimire, Sunil Kumar Sharma Dhakal, Pranil Rai, Nirvan Rai

**Affiliations:** 1 Department of General Surgery and Digestive Diseases, Nepal Mediciti Hospital, Bhaisepati, Lalitpur, Nepal

**Keywords:** *case report*, *computed tomography*, *obturator hernia*, *X-ray*

## Abstract

Encountering an obturator hernia itself is a rare entity for practicing surgeons globally. Synonymously known as the "old lady's hernia" is usually seen in fragile geriatric multiparous female patients. We share our experience of this rare entity where the patient presented with features of small bowel obstruction. Her diagnosis was delayed in the previously attended other centre as the possibility of an obturator hernia was overlooked. A timely clinical examination of the patient complemented by a computed tomography scan of the abdomen and pelvis helped us to obtain a diagnosis and proceed with immediate surgery. Intraoperatively the rarity was present bilaterally although the obstruction was on one side only. Prompt diagnosis and treatment benefits patients in this potentially lethal condition.

## INTRODUCTION

Obturator hernia (OH) is a rare entity that was first described by Professor Pierre Roland Arnaud de Ronsil with a reported incidence of 0.073% of all intraabdominal hernias in the West and 1% in the far East.^1,2^ Synonymously OH is also known as the "little old lady's hernia" and is six to nine times more common in emaciated, multiparous women in their seventh decade or older with a high mortality rate of up to 25%.^3^ In this case, low threshold for doing early computed tomography scan easily led us to the diagnosis of OH instantly and this rarity was present bilaterally in the patient.

## CASE REPORT

A 76 years old lady, a mother of six children presented with a complaint of pain in the abdomen associated with multiple episodes of vomiting for 3 days where she was evaluated and discharged home with oral medications. However, her symptoms persisted and she presented at the emergency of Nepal Mediciti Hospital with complaints of increasing abdominal pain associated with feculent vomiting, progressively increasing abdominal distension and obstipation. She also gave a history of chronic cough with heavy biofuel exposure. She had a kyphotic posture but she was unaware of the cause of her kyphosis. She didn't have a significant medical family or psycho-social history.

She had no previous history of abdominal surgery.

**Figure 1:**
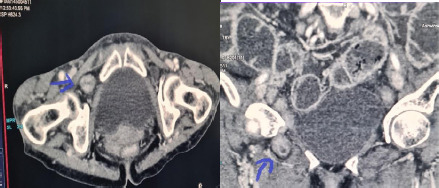
The axial and coronal images of the CT scan showing right sided obturator hernia containing a bowel loop (marked by an arrow) causing an obstruction.

The patient weighed 38 kg. She was tachycardiac, hypotensive, slightly short of breath and dehydrated. Her abdomen was grossly distended with diffuse tenderness and hyperactive bowel sounds were heard on auscultation. She didn't have groin swelling and per rectal examination was unremarkable. The patient was resuscitated with two litres of lactated ringer solution and her urine output was monitored with a Foley catheter. A nasogastric tube was inserted which yielded feculent effluent. Her lab reports showed leukocytosis with neutrophilia and elevated urea levels. Other blood parameters were normal. An urgent contrast-enhanced computed tomography (CECT) scan of the abdomen and pelvis was done which showed a rightsided obturator hernia containing a small bowel loop lying between the pectineus and obturator muscles with thickened wall and narrowed lumen causing bowel obstruction (Figure 1).

**Figure 2:**
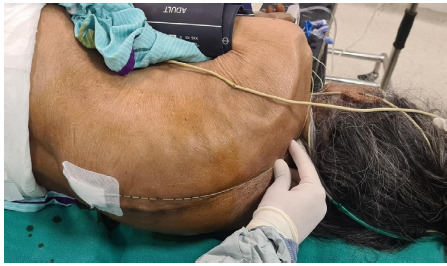
Epidural catheter inserted for postoperative pain management.

As the patient was discharged from another centre with oral medication, trying to convince them for the surgery was really challenging. The patient party was reluctant to undergo surgery and after a thorough counselling informed consent was taken from the patient's family and an urgent midline laparotomy was done. Our expert team of anaesthesiologists placed an epidural catheter despite her kyphotic posture which played an important role in the non-turbulent pain-free postoperative period (Figure 2).

**Figure 3:**
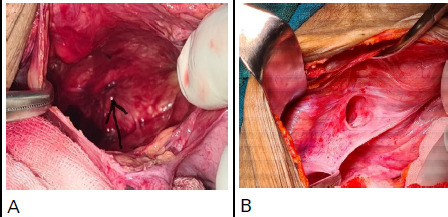
A) Right-sided obturator defect revealed after reduction of herniated bowel loop, B) Leftsided obturator hernia with empty sac.

The low midline laparotomy revealed that a nonischemic ileal loop had herniated in the right obturator canal causing upstream dilatation of small bowel loops. Along with that she also had an obturator hernia with an empty sac on the left side (Figure 3).

**Figure 4:**
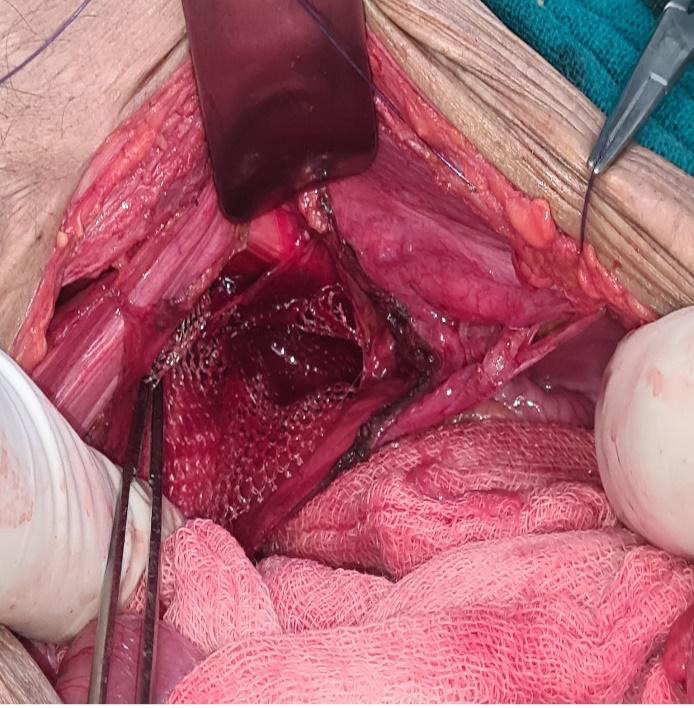
Mesh plug used to close the hernial defect.

The herniated viable ileal loop in the hernia was reduced into the peritoneal cavity and both the hernial defects were closed bilaterally with a mesh plug (Figure 4).

She was kept in the intensive care unit for 2 days and oral feeding was started on the second postoperative day. Her leukocyte count and renal function returned to normal range on the second postoperative day. Post-operative period was uneventful. She came out unscathed and was discharged on the 6^th^ postoperative day. At the time of discharge, she was tolerating oral feeding and was comfortable with oral analgesics. She followed up in the outpatient clinic a week later, where her recovery was found to be satisfactory.

## DISCUSSION

Obturator hernia (OH) is a rare condition encountered by a surgeon and accounts for less than 1% of all intraabdominal hernias.^2^ This is the only case encountered by our team since the inception of the hospital in 2017. This also shows the rarity of OH in our population. A study was published on a decade-long review of the emergency presentation of OH in Sydney, Australia which encountered just eighteen cases with a mortality rate of 27.8%. The women with a broader pelvis, greater transverse diameter and high laxity of pelvic floor secondary to their parity are more preponderant (about 6 to 9 times more than men) to develop this rare entity.^4^ It is less common on the left side due to the protection offered by the sigmoid colon to the obturator canal. However OH can be bilateral in 20% of the cases.^5^

Depending upon the type of surgery opted by the surgeon the rate of detection of occult contralateral hernia has been reported from 6% to 63%.^6^ Adhering to these findings in the literature our patient was an elderly, emaciated, multiparous female with a symptomatic OH on the right side and occult OH on the left.

Clinical diagnosis of OH is quite challenging due to the vague symptoms at presentation. In most cases, patients present with non-specific aching or dull abdominal pain associated with nausea and vomiting. Up to 80% of patients with OH usually have symptoms of intermittent partial bowel obstruction due to a high proportion exhibiting Richter's herniation of the bowel into the obturator canal.^7^ This is an important factor to be considered in the history of the patient to suspect the possibility of OH. The Howship-Romberg sign (pain on the medial aspect of the thigh on extension, adduction, or medial rotation of the hip) or Hannington - Kiff sign (loss of adductor reflex) is considered pathognomonic of irritation of the obturator nerve by the hernia but is rarely elicited in the emergency as the patient is usually sick.^8^

Early diagnosis of OH can be made with a computed tomography scan of the abdomen and pelvis and images showing bowel herniating through the obturator foramen and lying between the pectinus and obturator muscles. This has been shown to be the best diagnostic clue.^9,10^ We also opted for the immediate CT scan which aided in prompt diagnosis and appropriate treatment within no time. Various techniques have been advocated for the reduction of OH and repair of defects which may be intraperitoneal or extra peritoneal approaches along with or without mesh hernioplasty. Intraperitoneal approach includes open laparotomy or laparoscopic transabdominal preperitoneal repair. Extraperitoneal approach consists of either trans - inguinal approach or laparoscopic totally extraperitoneal approach.^6^ The intraperitoneal approach has the advantage of evaluating the bowel and treating a possible ischemic bowel with resection, especially in emergency cases. While the extraperitoneal approach is better for elective cases but the viability of herniated bowel has to be proven preoperatively.^6^ We opted for the intraperitoneal approach with a low midline laparotomy as our patient presented in the emergency with complete obstruction and a CT scan revealed that the bowel loops were grossly dilated with the possibility of bowel strangulation. We have planned to offer the laparoscopic extraperitoneal approach to the patients whenever we see them again as we have been routinely doing laparoscopic extraperitoneal surgeries for elective groin hernias.

To conclude, although obturator hernia is a rare entity it should always be thought of especially in the emaciated elderly lady without prior abdominal surgery who presents with features of intestinal obstruction. Detailed history and physical examination combined with an early CT scan could be very helpful to establish the preoperative diagnosis. Early diagnosis and prompt surgical treatment are the keys to reduce morbidity and mortality associated with this condition.
